# Identifying erlotinib-sensitive non-small cell lung carcinoma tumors in mice using [^11^C]erlotinib PET

**DOI:** 10.1186/s13550-014-0080-0

**Published:** 2015-02-12

**Authors:** Galith Abourbeh, Batel Itamar, Olga Salnikov, Sergey Beltsov, Eyal Mishani

**Affiliations:** Cyclotron-Radiochemistry-MicroPET Unit, Hadassah Hebrew University Hospital, Jerusalem, 91120 Israel

**Keywords:** [^11^C]Erlotinib, PET, Imaging, NSCLC, EGFR, TKI

## Abstract

**Background:**

Non-small cell lung carcinoma (NSCLC) represents approximately 80% of lung cancer cases, and over 60% of these tumors express the epidermal growth factor receptor (EGFR). Activating mutations in the tyrosine kinase (TK) domain of the EGFR are detected in 10% to 30% of NSCLC patients, and evidence of their presence is a prerequisite for initiation of first-line therapy with selective TK inhibitors (TKIs), such as gefitinib and erlotinib. To date, the selection of candidate patients for first-line treatment with EGFR TKIs requires an invasive tumor biopsy to affirm the mutational status of the receptor. This study was designed to evaluate whether positron emission tomography (PET) of NSCLC tumor-bearing mice using [^11^C]erlotinib could distinguish erlotinib-sensitive from erlotinib-insensitive or erlotinib-resistant tumors.

**Methods:**

Four human NSCLC cell lines were employed, expressing either of the following forms of the EGFR: (i) the wild-type receptor (QG56 cells), (ii) a mutant with an exon 19 in-frame deletion (HCC827 cells), (iii) a mutant with the exon 21 L858R point mutation (NCI-H3255 cells), and (iv) a double mutant harboring the L858R and T790M mutations (NCI-H1975 cells). Sensitivity of each cell line to the anti-proliferative effect of erlotinib was determined *in vitro. In vivo* PET imaging studies following i.v. injection of [^11^C]erlotinib were carried out in nude mice bearing subcutaneous (s.c.) xenografts of the four cell lines.

**Results:**

Cells harboring activating mutations in the EGFR TK domain (HCC827 and NCI-H3255) were approximately 1,000- and 100-fold more sensitive to erlotinib treatment *in vitro*, respectively, compared to the other two cell lines. [^11^C]Erlotinib PET scans could differentiate erlotinib-sensitive tumors from insensitive (QG56) or resistant (NCI-H1975) tumors already at 12 min after injection. Nonetheless, the uptake in HCC827 tumors was significantly higher than that in NCI-H3255, possibly reflecting differences in ATP and erlotinib affinities between the EGFR mutants.

**Conclusions:**

[^11^C]Erlotinib imaging in mice differentiates erlotinib-sensitive NSCLC tumors from erlotinib-insensitive or erlotinib-resistant ones.

## Background

The mortality from lung cancer is high and accounts for almost 30% of cancer-related deaths. Though the overall death rate from lung cancer is declining, the prognosis of stage IV non-small cell lung carcinoma (NSCLC) patients is poor, with a 5-year survival rate lower than 5% [1,2].

NSCLC represents approximately 80% of lung cancer cases, and over 60% of these tumors express the epidermal growth factor receptor (EGFR), rendering it a primary protein of interest of different targeted therapeutic approaches [1,3-5].

Alterations in the tyrosine kinase (TK) domain of the EGFR, such as the L858R point mutation and the E746-A750 deletion mutation, occur in 10% to 15% of Caucasian and 30% to 50% of Asian NSCLC patients [6]. These mutations are associated with a distinct pattern of EGFR downstream signaling and confer ‘oncogene addiction’, i.e., dependence of cells upon EGFR activity. Thus, in the presence of these sensitizing mutations, inhibition of EGFR signaling is detrimental to the cells [7-9].

Low molecular weight TK inhibitors (TKIs) of the EGFR, such as gefitinib and erlotinib, have been proven particularly effective as first-line treatment in NSCLC patients, whose tumors harbor activating mutations in the EGFR TK domain. Specifically, in the presence of such mutations, treatment with a selective EGFR TKI is associated with superior progression-free survival (PFS), increased overall response rate (ORR), a more favorable toxicity profile, and an improved quality of life with respect to cytotoxic chemotherapy [1,6,10,11]. Moreover, first-line treatment of mutation-negative NSCLC patients using selective EGFR TKIs is harmful and worsens PFS. Accordingly, EGFR TKIs are recommended as first-line treatment only for patients whose tumors harbor activating EGFR TK mutations [1,10].

Despite the significant improvement in PFS and ORR, all patients treated with EGFR TKIs inevitably develop resistance. Emergence of a secondary mutation in the TK domain of the receptor, such as the T790M point mutation, is the most common mechanism of resistance to EGFR TKIs, accounting for approximately 50% of patients, whose disease had progressed following treatment with TKIs [1,6,12,13].

At present, identification of candidate NSCLC patients for first-line treatment with TKIs requires screening for the existence of mutations, using tumor genotyping techniques and/or immunohistochemistry (IHC) [1,14]. These approaches require invasive procedures for tissue biopsies, which are not always readily accessible for sampling, could be of insufficient quality, and require time for mutation analysis. Furthermore, owing to the heterogeneity of tumors *vis-à-vis* the mutational status of EGFR, the data obtained from tissue samples does not necessarily reflect that of the entire primary tumor and is of limited value in predicting the molecular characteristics of distant metastases.

These hurdles have urged the pursuit of alternative, non-invasive approaches for evaluating and quantifying the mutational status of EGFR [15,16]. The use of a non-invasive imaging technique, such as positron emission tomography (PET), for identifying the mutational status of the EGFR TK in tumors should facilitate patient stratification prior to initiation of treatment with TKIs. Moreover, since approximately 50% of NSCLC patients treated with TKIs ultimately develop secondary mutations in the EGFR TK domain in tumors and consequently resistance to erlotinib treatment, PET should also afford longitudinal monitoring of EGFR mutational status in tumors.

During the past decade, numerous radiolabeled EGFR-targeted agents, namely antibodies and TKIs, have been investigated as probes for visualizing and quantifying EGFR expression in tumors using nuclear imaging modalities, such as single photon emission computed tomography (SPECT) and PET [16-29]. Notably, both erlotinib and gefitinib have been labeled with positron-emitting isotopes and evaluated in preclinical animal models. Reports on ^11^C- and ^18^F-labeled gefitinib imaging in tumor-bearing mice indicated that [^11^C]gefitinib has more potential than its fluorine-18-labeled congener, although to date, neither has progressed into clinical trials [26,29]. Conversely, reports on [^11^C]erlotinib have revealed its added value in imaging EGFR mutant-positive tumors not only in mice [19,24], but also in humans [18,20,28]. Hitherto, elevated tumor uptake of [^11^C]erlotinib has been demonstrated only in tumors harboring EGFR exon 19 deletions compared to tumors without activating EGFR mutations [18,19,24,28]. However, the extent to which [^11^C]erlotinib PET could identify NSCLC tumors that harbor other commonly detected TK mutations, such as the activating exon 21 L858R point mutation and the T790M gate-keeper mutation, which confers resistance to TKI therapy, has not been reported.

In the current study, we sought to further explore the potential of [^11^C]erlotinib in differentiating erlotinib-sensitive tumors from erlotinib-insensitive or erlotinib-resistant ones. To this end, four different human NSCLC cell lines were employed, two of which express the commonly encountered mutations in the EGFR TK domain (delE746-A750 mutation and L858R point mutation) and two additional lines expressing the secondary T790M mutation or wild-type EGFR (wtEGFR). [^11^C]Erlotinib PET/CT scans were carried out in athymic nude mice grafted with subcutaneous (s.c.) xenografts of these tumor cell lines. The presented results indicate that [^11^C]erlotinib scans could distinguish NSCLC tumors that express activating mutations in the EGFR TK domain and are sensitive to erlotinib treatment, from tumors that harbor wtEGFR or the double-mutated (L858R + T790M) receptor and do not respond to erlotinib therapy. This data further substantiate the potential of [^11^C]erlotinib PET as a non-invasive tool to identify NSCLC patients who are most likely to benefit from treatment with TKIs and to monitor the mutational status of EGFR during the course of treatment.

## Methods

### General

Insulin, transferrin, HEPES, and sodium pyruvate were purchased from Biological Industries (BI) (Kibbutz Beit Haemek, Israel). Sodium selenite, hydrocortisone, ethanolamine, O-phosphorylethanolamine, 3,3′,5-triiodo-l-thyronine (T_3_), and bovine serum albumin (BSA) were purchased from Sigma-Aldrich (Rehovot, Israel). Recombinant human EGF was purchased from PeproTech Asia (Rehovot, Israel).

Hsd:Athymic Nude-Fox1nu mice (male, 4 to 5 weeks) were obtained from Harlan (Rehovot, Israel). All animal studies were conducted under a protocol approved by the Animal Research Ethics Committee of the Hebrew University of Jerusalem and in accordance with its guidelines. Animals were allowed to acclimate for at least 3 days, prior to their inoculation with tumor cells, and were routinely kept in 12-h light/dark cycles and provided with food and water *ad libitum*.

### Cell culture

The following human NSCLC cell lines were employed: QG56, HCC827, NCI-H1975, and NCI-H3255. The latter were purchased from the National Cancer Institute - Division of Cancer Treatment and Diagnosis (NCI-DCTD) tumor repository (Frederick, MD, USA) and were regularly maintained in ACL-4 medium, containing insulin (0.02 mg/mL), transferrin (0.01 mg/mL), sodium selenite (25 nM), hydrocortisone (50 nM), EGF (1 ng/mL), ethanolamine (0.01 mM), O-phosphorylethanolamine (0.01 mM), triiodothyronine (100 pM), BSA (0.2% (*w*/*v*)), HEPES (10 mM), sodium pyruvate (0.5 mM), and l-glutamine (2 mM) in RMPI-1640 medium (Invitrogen™, Life Technologies, Carlsbad, MA, USA). HCC827 and NCI-H1975 cells were maintained in RMPI-1640 (#30-2001, ATCC), and QG56 were grown in RPMI-1640 (Invitrogen™) at 37°C in a humidified atmosphere of 95% air and 5% CO_2_. All media were supplemented with fetal bovine serum (FBS, 10%) and antibiotics (penicillin 10^4^ units/L, streptomycin 10 mg/L) (BI, Israel).

### Inhibition of cell growth

QG56 (4,000 cells), HCC827, NCI-H3255 (5,000 cells), and NCI-H1975 (7,000 cells) were cultured in 96-well plates. After 24 to 48 h, cells were treated with increasing concentrations (0 to 100 μM) of erlotinib (Cayman Chemical Company, Ann Arbor, MI, USA). The media containing erlotinib (0.05% DMSO, 0.1% ethanol) were freshly prepared and replaced every 24 h. Following 72 h of treatment, cell growth was determined by methylene blue assay [30]. The median inhibitory concentration (IC_50_) of erlotinib for cell growth of each cell line was calculated using GraphPad Prism 5.0 software. Experiments were repeated thrice for each cell line, in three to six replicates per tested concentration.

### NSCLC xenografts

Mice were anesthetized with isoflurane (1% to 2% in oxygen) and injected s.c. in each front flank with a suspension of five million cells in a medium containing Matrigel (BD Biosciences, Beit Haemek, Israel, 20% (*v*/*v*)).

### Western blot

Cell lysates and tumor tissue extracts were prepared in cold (0°C to 4°C) modified RIPA buffer [31], supplemented with protease inhibitor cocktail for mammalian tissues (P8340, Sigma, 1%). Equal amounts of each sample (30 μg of total protein) were loaded and separated by SDS-PAGE (10%). Proteins were electrophoretically transferred to a nitrocellulose membrane, and the latter was blocked in 3% BSA in TBST buffer (50 mM Tris-HCl, pH 7.5, 0.1% Tween 20, and 150 mM NaCl) for 30 min. Corresponding parts of the membrane were incubated overnight (4°C with gentle shake) with either of the following primary antibodies, diluted in 3% BSA/TBST: (1) rabbit polyclonal EGFR 1005 antibody (sc-03, Santa Cruz Biotechnology Inc., Dallas, TX, USA), (2) mouse monoclonal phosphotyrosine PY20 antibody (sc-508, Santa Cruz Biotechnology Inc.), or (3) mouse monoclonal anti-β-actin antibody (mAbcam 8224, Abcam, Cambridge, UK). The membrane parts were washed thoroughly with TBST buffer and incubated for 1 h with the corresponding horseradish peroxidase-conjugated IgGs (Santa Cruz Biotechnology Inc.) in 3% BSA/TBST. Finally, the membranes were washed in TBST, and immunoreactive proteins were visualized using the EZ-ECL kit (BI, Israel). Densitometry was performed using TINA 2.10 g software, and the intensity of each EGFR band was normalized to that of the corresponding β-actin band to correct for possible differences in the content of total loaded protein.

### [^11^C]Erlotinib synthesis

The radiosynthesis of [^11^C]erlotinib was based on a previously published procedure [19,20], with slight modifications. [^11^C]CO_2_ (50.5 ± 2 GBq (*n* = 25)) was trapped at −160°C. Subsequently, the temperature of the cooling trap was increased to −5°C, and the activity was transferred by a stream of argon (40 mL/min) into the first reactor at −50°C, containing 300 μL of 0.25 N lithium aluminum hydride (ABX, Radeberg, Germany) in dry tetrahydrofuran. After 2.5 min, the solvent was removed under reduced pressure, and the reactor temperature was increased to 160°C. Next, hydroiodic acid (0.5 mL, Merck, White House Station, NJ, USA) was added, and [^11^C]CH_3_I was distilled through a NaOH column (Merck) under argon flow (25 mL/min) and transferred into a second reactor at −15°C, containing 10 mg of 6-*O*-desmethyl erlotinib (OSI-774, Selleck Chemicals, Houston, TX, USA) dissolved in 0.3 mL *N*,*N*-dimethylformamide, containing 3.5 mg of sodium hydride. At the end of the 1-min distillation step, 20.6 ± 1 GBq (*n* = 25) was trapped in the second reactor. The reactor was sealed and heated to 90°C for 1.5 min. Then, the reactor temperature was increased to 120°C for an additional 5 min. Following a 6.5-min reaction, volatiles were removed under argon flow (at reduced pressure) at a temperature of 90°C. The mixture was cooled to 40°C, 0.6 mL of acetonitrile/water was added, and the crude product was injected into a semi-preparative HPLC column, equipped with a variable wavelength UV detector (254 nm) and a radioactivity detector with NaI crystals. A Phenomenex C18 column (5 μm, 10 mm × 250 mm; Torrance, CA, USA) was used, with a mobile phase system of acetonitrile:acetate buffer 0.1 M, pH 3.8 (4:6), at a constant flow rate of 4 mL/min. The retention time of [^11^C]erlotinib was 10.5 min, and the product was collected into a flask containing 24 mL HPLC water and 350 μL of 1 M NaOH. Subsequently, the solution was passed through a Sep-Pak Plus C18 cartridge (Waters Corporation, Milford, MA, USA), which was pre-activated with 5 mL EtOH, and washed with 10 mL HPLC water prior to the synthesis. The cartridge was washed with 4 mL of water, and [^11^C]erlotinib was eluted using 1.3 mL of EtOH followed by 11 mL of sterile isotonic saline (B. Braun, Melsungen, Germany). Quality control analysis was performed on an analytical HPLC, equipped with a variable wavelength UV detector (254 nm) and a radioactivity detector with NaI crystals. A Phenomenex C18 column (5 μm, 4.6 mm × 250 mm) was used, with a mobile phase system of acetonitrile:acetate buffer 0.1 M, pH 3.8 (37:63) for 30 min, at a constant flow rate of 1 mL/min.

### MicroPET/CT studies

Tumor-bearing mice (30 ± 1 g (*n* = 27)) were anesthetized with isoflurane (1% to 2.5% in O_2_) and maintained normothermic using a heating pad. Following a CT attenuation-correction scan, PET acquisitions were carried out in list mode using an Inveon™ MM PET-CT small-animal dedicated scanner (Siemens Medical Solutions, Malvern, PA, USA). PET scans were started at the time of [^11^C]erlotinib injection via the lateral tail vein (16.36 ± 0.6 MBq (*n* = 27)) and lasted for 1 h. Subsequently, mice were maintained at the same position and injected i.v. with [^18^F]FDG (5.67 ± 0.2 MBq (*n* = 24)). After a 40-min uptake period, a second 20-min PET acquisition was performed. Carrier-added studies were carried out in HCC827 tumor-bearing mice (*n* = 5), wherein erlotinib (6.7 mg/kg, dissolved in Cremophor EL/ethanol/saline (1:1:8)) was co-injected with the radiolabeled compound.

Emission sinograms were normalized and corrected for attenuation, scatter, randoms, dead time, and decay. Image reconstruction was performed using Fourier rebinning and two-dimensional ordered-subsets expectation maximization (2D-OSEM), with a voxel size of 0.776 × 0.776 × 0.796 mm^3^. Image analysis and quantification were performed using Inveon Research Workplace 4.2 (Siemens Medical Solutions). Delineation of tumors' volumes of interest (VOIs) was performed by manual segmentation, based on the fused [^18^F]FDG and CT images, and the corresponding [^11^C]erlotinib time-activity curves (TACs) were calculated. Distribution of activity was calculated as the percentage of injected dose per milliliter of tissue (%ID/mL). Standardized uptake values (SUVs) were calculated as the product of %ID/mL and the total body weight of the animal.

### Statistics

Statistical analysis was made using GraphPad Prism 5 software. Unless otherwise stated, data is expressed as mean ± SEM. Comparisons of [^11^C]erlotinib uptake in tumors in imaging studies were made using one-way ANOVA, followed by the *Bonferroni* post hoc test. The level of significance was regularly set at *p* < 0.05.

## Results

### Inhibition of cell growth

**Figure 1:**
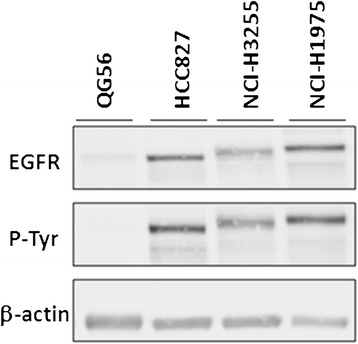
**Representative Western blots of four human NSCLC cell lysates comparing the extent of EGFR and phospho-EGFR expression.** β-actin served as a reference for equal loading.

Four human NSCLC cell lines expressing different forms of the EGFR were investigated. Sensitivity of each cell line to the anti-proliferative effect of erlotinib was evaluated by methylene blue assay and is presented in Table 1. Two cell lines, HCC827 and NCI-H3255, which express activating mutations in the EGFR, were erlotinib-sensitive, having mean IC_50_ values of 4 and 41 nM, respectively. Two additional cell lines, QG56 (expressing wtEGFR) and NCI-H1975 (expressing the L858R + T790M point mutations), were significantly less sensitive to erlotinib treatment, with mean IC_50_ values of 8.9 and 4.3 μM, respectively. Thus, *in vitro*, NCI-H3255 and HCC827 cells were 100- to 1,000-fold more sensitive to the anti-proliferative effect of erlotinib compared to QG56 and NCI-H1975 cells. In agreement with previous reports, Western blot analysis (Figure 1) indicated that the sensitivity to erlotinib treatment could not be attributed to differences in total EGFR or EGFR-associated phosphotyrosine (PY) levels, since neither of those correlated with erlotinib sensitivity of the tested cell lines [14,32,33].

**Table 1:** **Sensitivity of four human NSCLC cell lines to the anti-proliferative effect of erlotinib** ***in vitro***

**Cell line**	**Type of EGFR mutation**	**IC**_**50**_ **of erlotinib [μM]**	**Erlotinib sensitive**
QG56	None (wtEGFR)	8.9 ± 0.5***	No
HCC827	Activating (delE746-A750)	0.004 ± 0.0009	Yes
NCI-H3255	Activating (L858R point mutation)	0.041 ± 0.007	Yes
NCI-H1975	Double mutation (L858R + T790M point mutations)	4.3 ± 0.8***	No

***p < 0.001 with respect to HCC827 cells.

### [^11^C]Erlotinib synthesis

[^11^C]Erlotinib was obtained after an approximately 40-min synthesis, including formulation, with an average radioactivity of 2.42 ± 0.3 GBq (*n* = 25) in 12.3 mL of 10% EtOH/saline. The average specific activity at the end of synthesis was 26.14 ± 2.5 GBq/μmol (*n* = 25), and the radiochemical purity was above 97%.

### MicroPET/CT studies

**Figure 2:**
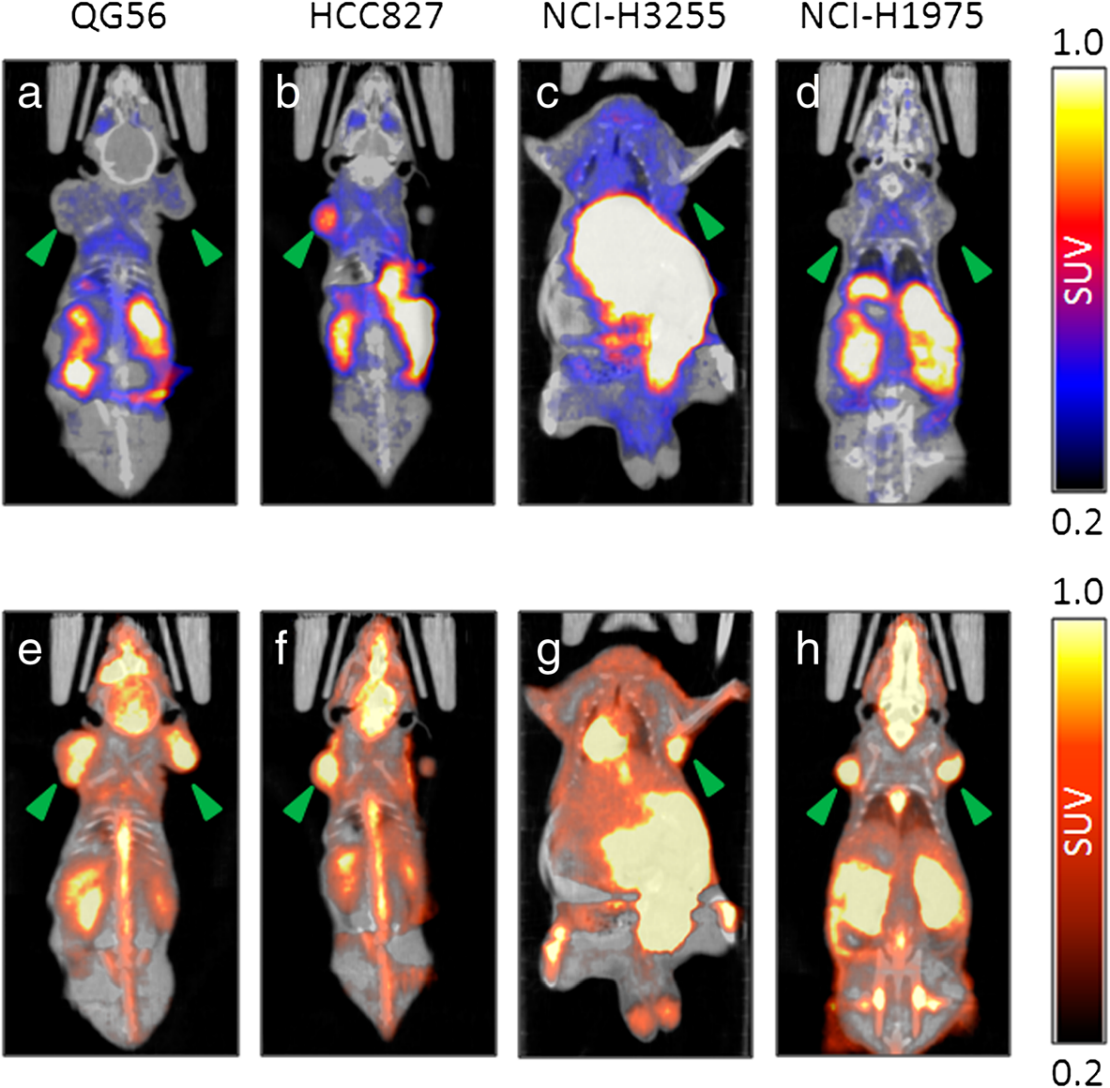
**Representative PET/CT slice images of NSCLC tumor-bearing mice.** The images were taken following sequential injections of [^11^C]erlotinib **(a-d)** and [^18^F]FDG **(e-h)** into each mouse, demonstrating (arrowheads) [^11^C]erlotinib uptake in erlotinib-sensitive tumors **(b, c)** and in erlotinib-insensitive ones **(a, d)**. [^11^C]Erlotinib and [^18^F]FDG images depict the summation of radioactivity uptake from 30 to 60 min and 40 to 60 min after injection, respectively. Each set of [^11^C]erlotinib and [^18^F]FDG images was normalized to the same scale.

PET acquisitions of tumor-bearing mice were carried out for 1 h following i.v. injection of [^11^C]erlotinib. Subsequently, mice were injected with [^18^F]FDG and scanned again 40 min after injection. The [^18^F]FDG scans served a dual purpose: to confirm tumor viability and to delineate the tumor's VOI, particularly in cases where [^11^C]erlotinib uptake in tumor was not prominent (Figure 2).

**Figure 3:**
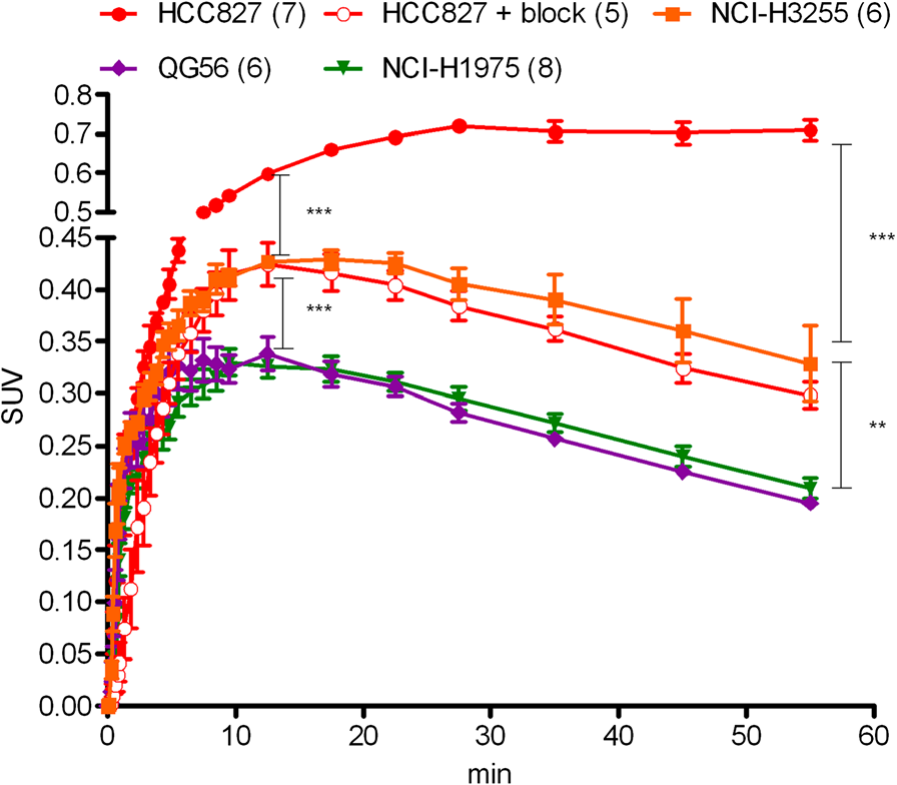
**TACs representing NSCLC tumor kinetics following injection of [** ^**11**^ **C]erlotinib.** Tumor TACs were obtained after i.v. injection of [^11^C]erlotinib into NSCLC tumor-bearing mice, demonstrating increased radioactivity uptake in erlotinib-sensitive tumors (HCC827 and NCI-H3255) compared to insensitive (QG56) or resistant (NCI-H1975) ones. The number of scanned tumors is indicated in brackets. **p < 0.01 and ***p < 0.001.

TACs representing the kinetics in tumors following [^11^C]erlotinib injection are presented in Figure 3, revealing distinct differences between erlotinib-sensitive and erlotinib-insensitive tumors. The uptake in both sensitive tumors, HCC827 and NCI-H3255, was significantly higher than that observed in QG56 and NCI-H1975 tumors, already at 12 min after injection and throughout the 60-min scan (Figure 3, Table 2). Thus, [^11^C]erlotinib could clearly distinguish erlotinib-sensitive tumors from insensitive ones. Nonetheless, whereas the uptake in HCC827 tumors was high and sustained (SUV > 0.6 starting 12 min after injection), the peak SUV in NCI-H3255 tumors was 0.43, at the same time point, further declining to 0.33 at 1 h (Figure 3). In addition, the uptake in HCC827 tumors was specific, as revealed in blocking studies, wherein more than 50% reduction in tumor uptake was measured after administration of non-labeled erlotinib in excess. Notably, the kinetics of [^11^C]erlotinib in HCC827 tumors in blocking studies was comparable to that measured in NCI-H3255 tumors without the addition of carrier in excess.

**Table 2:** **Tumor SUVs at early (12 min) and late (60 min) time points after [** ^**11**^ **C]erlotinib injection**

**Tumor type**	**Early SUV**	**Late SUV**
QG56	0.338 ± 0.039* (6)	0.195 ± 0.010**^,^ *** (6)
HCC827	0.598 ± 0.013 (4)	0.709 ± 0.069 (7)
HCC827 + block	0.424 ± 0.046** (5)	0.298 ± 0.029** (5)
NCI-H3255	0.427 ± 0.014** (6)	0.328 ± 0.089** (6)
NCI-H1975	0.326 ± 0.032* (8)	0.209 ± 0.028**^,^ *** (8)

Results are presented as mean ± SD, and the number of scanned tumors is indicated in brackets. *p < 0.001 with respect to HCC827 and NCI-H3255 tumors. **p < 0.001 with respect to HCC827 tumors. ***p < 0.01 with respect to NCI-H3255 tumors.

### Western blot

**Figure 4:**
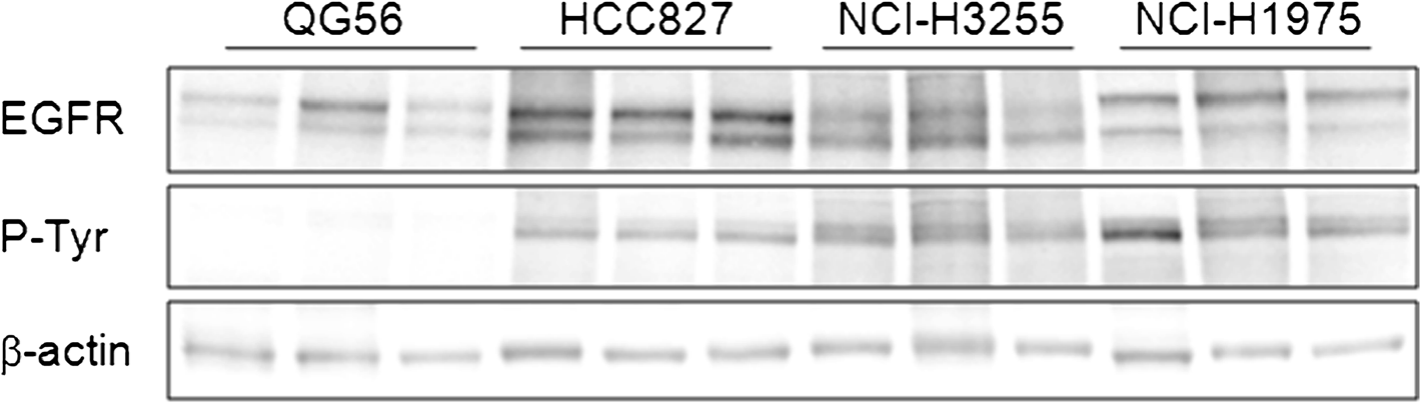
**Representative Western blots of NSCLC tumor extracts, demonstrating total EGFR and phospho-EGFR levels in tumors.** *n* = 3 per group. β-actin served as a reference for equal loading.

The relative expression of total EGFR and EGFR-associated PY levels in the four investigated NSCLC tumors was evaluated by Western blot analysis. The results illustrated in Figure 4 indicated HCC827 as those expressing the highest levels of EGFR, followed by NCI-H3255 tumors (approximately 73%), NCI-H1975 (approximately 65%), and QG56 tumors (approximately 43%). Moreover, the latter also had significantly lower EGFR-associated PY levels, compared to the other three tumors, wherein similar levels of EGFR-PY were detected.

## Discussion

Target-specific TKIs, peptides, and antibodies have paved the way to a more personalized treatment of cancer, wherein therapy is tailored based on the molecular characteristics of the patients' tumors rather than on tumor staging and histopathology alone [27,34-36]. Nuclear medicine imaging techniques, such as PET and SPECT, offer a highly sensitive and non-invasive means for detecting and quantifying the expression of molecular targets *in vivo*. Thus, upon development of a suitable radiolabeled pharmaceutical, one could potentially visualize and quantify the expression of specific target proteins in the entire body in a non-invasive, longitudinal manner, instead of relying on site-specific, invasive tissue sampling. This approach could not only simplify patient stratification and selection prior to initiation of targeted therapy, but could also facilitate treatment monitoring and enable prolonged measurement of changes in target expression [37].

Specific EGFR TKIs, such as gefitinib and erlotinib, gained FDA approval for the treatment of advanced NSCLC in 2003 and 2004, respectively. The clinical data gathered thus far indicates that only patients whose tumors harbor distinct activating mutations in the TK domain of the receptor are likely to benefit from first-line treatment with EGFR TKIs. These mutations, typically exon 19 in-frame deletions (approximately 45%) and the L858R point mutation in exon 21 (40% to 45%), are detected in 10% to 30% of NSCLC patients [38,39]. The relatively limited prevalence of these sensitizing mutations requires that the EGFR mutational status be verified prior to the initiation of first-line therapy with EGFR TKIs. To date, targeted mutation testing uses specific and sensitive methods, yet it requires tumor biopsy. This approach is invasive and lengthy with regard to the time required for mutation analysis and provides information about the sampled site alone. Moreover, it is not favorable for obtaining molecular information in the long term, as would be required during EGFR TKI therapy, owing to the development of secondary mutations in the receptor and subsequent resistance to TKI treatment [1,6].

The present work further highlights the potential of [^11^C]erlotinib PET in discriminating erlotinib-sensitive NSCLC tumors from erlotinib-insensitive or resistant tumors. This methodology should provide sufficient information *vis-à-vis* the potential clinical benefit of patients from EGFR TKI therapy, hopefully circumventing the need for invasive tissue biopsies.

Four human NSCLC tumors were employed in this study, expressing wtEGFR (QG56), the commonly encountered activating mutations in the TK domain of the receptor, namely the delE746-A750 mutation (HCC827) and the L858R point mutation (NCI-H3255) or the L858R + T790M mutations (NCI-H1975). The latter cell line was selected since secondary mutations in the TK domain, such as the T790M mutation, are encountered in approximately 50% of EGFR TKI-treated patients, who develop resistance to therapy [1,6,38]. The effect of erlotinib on cell proliferation was evaluated *in vitro* for each cell line, and the corresponding IC_50_ values were calculated, as presented in Table 1. The results indicate that HCC827 and NCI-H3255 cells were significantly more sensitive to erlotinib treatment compared to QG56 and NCI-H1975 cells, having mean IC_50_ values of 4 and 40 nM, compared to 8.9 and 4.3 μM, respectively.

Subsequently, [^11^C]erlotinib PET acquisitions of tumor-bearing mice were carried out in order to determine whether the differences between cell lines *vis-à-vis* erlotinib sensitivity *in vitro* could be translated into distinct patterns of [^11^C]erlotinib tumor uptake *in vivo*. Since [^11^C]erlotinib uptake in tumors was not always evident (Figure 2), an [^18^F]FDG PET acquisition was carried out following each [^11^C]erlotinib scan, as aforementioned. The TACs of [^11^C]erlotinib uptake in tumors are illustrated in Figure 3, revealing statistically significant higher uptake of [^11^C]erlotinib in erlotinib-sensitive tumors vs. erlotinib-insensitive or erlotinib-resistant tumors. This differential uptake was measured as early as 12 min after [^11^C]erlotinib injection and had lasted throughout the 60-min scan period. Interestingly, however, whereas the profile of [^11^C]erlotinib uptake in both QG56 and NCI-H1975 tumors was almost identical, the uptake of [^11^C]erlotinib in HCC827 tumors was notably different from that observed in NCI-H3255 tumors. Specifically, [^11^C]erlotinib uptake in HCC827 tumors was relatively high and persistent, with mean SUVs of 0.60 and 0.71 at 12 and 60 min, respectively, compared to corresponding mean SUVs of 0.43 and 0.33 for NCI-H3255 tumors (Table 2). Furthermore, the TACs obtained for NCI-H3255 tumors were highly similar to those of HCC827 after co-administration of non-labeled erlotinib in excess.

Several factors could account for the observed differences in both kinetics and absolute [^11^C]erlotinib uptake values in HCC827 vs. NCI-H3255 tumors. First, the overall concentration of the target protein (*B*_max_) is expected to affect the extent of ligand binding [24,40]. Western blot analysis of tumor lysates indicated approximately 30% lower levels of EGFR in NCI-H3255 tumors compared to HCC827 (Figure 4), consistent with the approximately 30% lower SUV of [^11^C]erlotinib in NCI-H3255 tumors at 12 min after injection, the time of peak radioactivity uptake in these tumors (Figure 3). Still, differences in EGFR expression in tumors per se are most likely not the main determinant of the overall observed differences in [^11^C]erlotinib tumor uptake. As was recently demonstrated, elevated expression of EGFR or phospho-EGFR, such as that observed in U87ΔEGFR glioma tumors, does not guarantee enhanced uptake of [^11^C]erlotinib [24]. Similarly, our results indicate that although higher levels of EGFR and phospho-EGFR were measured in NCI-H1975 compared to QG56 tumors (Figure 4), the uptake of [^11^C]erlotinib in the two xenografts was comparable (Figure 3, Table 2). Thus, the cumulative evidence points to the mutational status of the EGFR TK domain rather than the level of EGFR expression as the major factor affecting [^11^C]erlotinib uptake in tumors. Nonetheless, when comparing the uptake of [^11^C]erlotinib in tumors that harbor activating mutations in the EGFR TK domain, the expression levels of the receptor should come into play [24].

Then, different binding affinities of the EGFR TK mutants to both adenosine triphosphate (ATP) and erlotinib could explain the heterogeneous kinetics of [^11^C]erlotinib in the two erlotinib-sensitive tumors. Carey et al. have demonstrated that compared to wtEGFR, the delE746-A750 and L858R mutants had significantly lower binding affinities to ATP, with corresponding approximately 26- and approximately 2-fold higher Michaelis-Menten constants (*K*_M_) with respect to the wtEGFR [41]. At the same time, these mutants were also reported to hold higher affinities to erlotinib, with respective 5.3- and 2.8-fold lower inhibition constant values (*K*_i_), compared to the wild-type receptor. Consequently, tumors harboring the delE746-A750 mutation would be expected to bind [^11^C]erlotinib more avidly than those expressing the L858R mutation.

**Figure 5:**
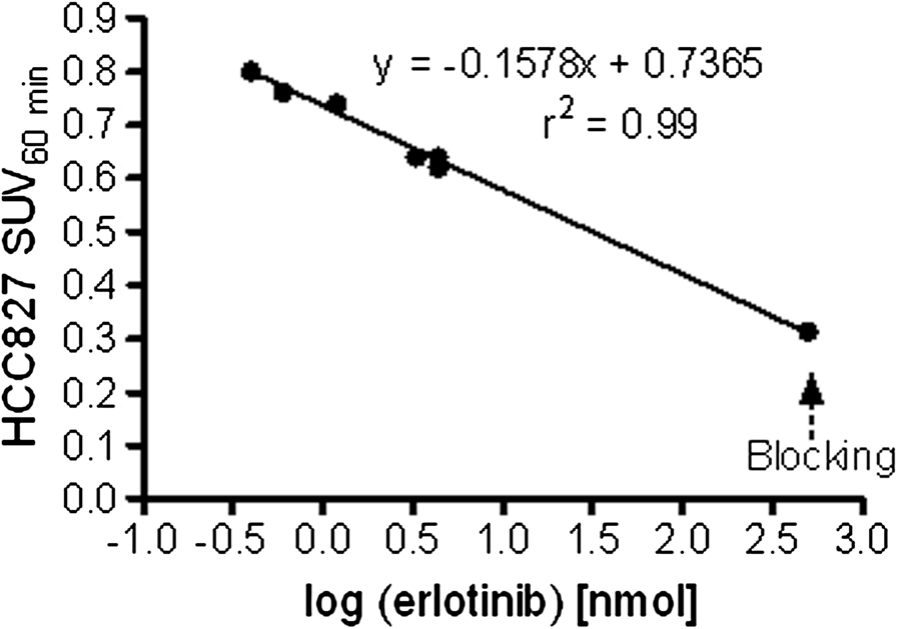
**Inverse correlation between [** ^**11**^ **C]erlotinib uptake in HCC827 tumors at 60 min after injection and injected carrier mass.** *n* = 12. The last point on the right represents the administered blocking dose (6.7 mg/kg, *n* = 5).

Increased uptake of [^11^C]erlotinib in tumors harboring the delE746-A750 mutation compared to wtEGFR has been demonstrated in both NSCLC patients [18] and mouse models [19,24]. The present results are in good agreement with those published by Petrulli et al. [24], with respect to the sustained uptake of [^11^C]erlotinib in HCC827 tumors, albeit lower mean SUVs were measured in the current study (0.7 vs. approximately 1.3 at 60 min after injection). The smaller tumor uptake values measured in this study could partly be attributed to the lower specific activity of [^11^C]erlotinib obtained here (26.14 ± 2.5 GBq/μmol), compared to that published by Petrulli and colleagues (159.1 ± 48 MBq/nmol) [24], resulting in a higher injected mass of non-labeled carrier. Based on the results of the present research, a proposed correlation between the injected erlotinib mass and the SUV of HCC827 tumors at 60 min after injection (SUV_60 min_) is presented in Figure 5, suggesting that the relationship between the two is exponential rather than linear. Thus, following injection of an 11-fold higher carrier mass (4.4 vs. 0.4 nmol), the SUV_60 min_ of [^11^C]erlotinib in HCC827 tumors had decreased by approximately 21% (from 0.8 to 0.63). Consistent with this notion, an excess non-labeled erlotinib dose of approximately 500 nmol, such as that administered in this study, should result in an SUV_60 min_ of approximately 0.3 for HCC827 tumors, as was indeed obtained in the blocking experiments (Figure 5). Moreover, if the proposed relation between the injected carrier mass and the SUV_60 min_ of HCC827 tumors is valid, increasing the blocking dose from 6.7 mg/kg to approximately 30 mg/kg should further reduce the SUV_60 min_ of HCC827 to approximately 0.2, a value lower than the measured SUV_60 min_ for NCI-H3255 tumors (0.33) and similar to that obtained in QG56 and NCI-H1975 tumors (0.2) (Figure 3, Table 2). This possibility is under investigation.

In the current study, calculation of [^11^C]erlotinib uptake in tumors relied on SUV measurements rather than on kinetic parameters, such as the volume of distribution (*V*_T_) or the binding potential (BP). Compared to SUV, which does not distinguish target-specific from non-specific binding, *V*_T_ and BP reflect the specific binding and are therefore more sensitive measures of *B*_max_. Nonetheless, the ability to distinguish significant differences in [^11^C]erlotinib uptake between erlotinib-sensitive and erlotinib-resistant tumors using SUVs suggests that a more pronounced difference would have been observed had a kinetic analysis been performed, and provides an initial proof-of-concept prior to the validation by full kinetic analysis.

In addition to [^11^C]erlotinib, the use of 3′-deoxy-3′-[^18^F]fluorothymidine ([^18^F]FLT) PET as a non-invasive tool for identifying TKI-responsive NSCLC tumors has been reported in preclinical mouse models and in humans [42-45]. Commonly, these studies measured the change in [^18^F]FLT tumor uptake prior to and after initiation of EGFR TKI therapy, to predict response to treatment. Albeit less straightforward than [^11^C]erlotinib scans, this approach has successfully differentiated TKI-sensitive NSCLC tumors from unresponsive ones. Notably, [^18^F]FLT PET scans in NSCLC tumor-bearing mice have demonstrated their utility in identifying TKI-resistant tumors, which harbor the most frequently encountered mutations associated with TKI resistance, i.e., the secondary T790M mutation [44] and MET amplification [43]. In this study, the potential of [^11^C]erlotinib PET in differentiating TKI-resistant tumors that contain the T790M mutation from TKI-sensitive tumors has been demonstrated. The utility of this approach to identify TKI-resistant NSCLC tumors with MET amplification warrants further investigation.

## Conclusions

The present study illustrates the usage of [^11^C]erlotinib PET for diagnosing the commonly encountered EGFR TK mutations in NSCLC patients and for discriminating erlotinib-sensitive tumors from erlotinib-insensitive or erlotinib-resistant tumors. To the best of our knowledge, this is the first demonstration of increased [^11^C]erlotinib uptake in NSCLC tumors harboring the L858R point mutation. Overall, these data further corroborate the potential of [^11^C]erlotinib PET in identifying patients who are likely to benefit from EGFR TKI therapy.
